# Urine Proteomics Differentiate Primary Thrombotic Antiphospholipid Syndrome From Obstetric Antiphospholipid Syndrome

**DOI:** 10.3389/fimmu.2021.702425

**Published:** 2021-08-19

**Authors:** Zhuochao Zhou, Yijun You, Fan Wang, Yue Sun, Jialin Teng, Honglei Liu, Xiaobing Cheng, Yutong Su, Hui Shi, Qiongyi Hu, Huihui Chi, Jinchao Jia, Liyan Wan, Tingting Liu, Mengyan Wang, Ce Shi, Chengde Yang, Junna Ye

**Affiliations:** ^1^Department of Rheumatology and Immunology, Ruijin Hospital, Shanghai Jiao Tong University School of Medicine, Shanghai, China; ^2^Department of Laboratory Medicine, Ruijin Hospital, Shanghai Jiao Tong University School of Medicine, Shanghai, China

**Keywords:** thrombotic antiphospholipid syndrome, obstetric antiphospholipid syndrome, CXCL12, PDGFB, urine proteomics

## Abstract

Antiphospholipid syndrome (APS) is a multisystem disorder characterized by thrombosis and/or recurrent fetal loss. This clinical phenotype heterogeneity may result in differences in response to treatment and prognosis. In this study, we aimed to identify primary thrombotic APS (TAPS) from primary obstetric APS (OAPS) using urine proteomics as a non-invasive method. Only patients with primary APS were enrolled in this study from 2016 to 2018 at a single clinical center in Shanghai. Urine samples from 15 patients with TAPS, 9 patients with OAPS, and 15 healthy controls (HCs) were collected and analyzed using isobaric tags for relative and absolute quantification (iTRAQ) labeling combined with liquid chromatography-tandem mass spectrometry analysis to identify differentially expressed proteins. Cluster analysis of urine proteomics identified differentiated proteins among the TAPS, OAPS, and HC groups. Urinary proteins were enriched in cytokine and cytokine receptor pathways. Representative secreted cytokines screened out (fold change >1.20, or <0.83, *p*<0.05) in these differentiated proteins were measured by enzyme-linked immunosorbent assay in a validation cohort. The results showed that the levels of C-X-C motif chemokine ligand 12 (CXCL12) were higher in the urine of patients with TAPS than in those with OAPS (*p*=0.035), while the levels of platelet-derived growth factor subunit B (PDGFB) were lower in patients with TAPS than in those with OAPS (*p*=0.041). In addition, correlation analysis showed that CXCL12 levels were positively correlated with immunoglobulin G anti-β2-glycoprotein I antibody (*r*=0.617, *p*=0.016). Our results demonstrated that urinary CXCL12 and PDGFB might serve as potential non-invasive markers to differentiate primary TAPS from primary OAPS.

## Introduction

Antiphospholipid syndrome (APS) is a multisystem disorder characterized by a combination of arterial and/or venous thrombosis, recurrent fetal loss in women, and persistent presence of antiphospholipid antibodies (aPLs) including lupus anticoagulant (LAC), anti-β2-glycoprotein I (aβ2GPI) and anti-cardiolipin antibody (aCL) (1). APLs constitute a heterogeneous group of immunoglobulins directed against phospholipids or specific phospholipid-binding plasma proteins (2). The persistent presence of aPLs is recognized as an important laboratory diagnostic criterion for the definite classification of patients with APS according to the updated 2006 Sydney modification ‘Sapporo criteria’ (3). APS is a heterogeneous disease combined with thrombotic and obstetric complications (4). Genomic and proteomic studies have been carried out to explore the underlying mechanisms. For example, monocytes derived from patients with APS, patients with thrombosis without APS, and healthy controls (HCs) expressed different genes, such as annexin I and annexin II (5). Furthermore, IgG from patients with only thrombosis and only obstetric complications triggered different signaling pathways in monocytes (6). Another study also reported that being treated with thrombotic or obstetric APS IgG compared with HC IgG, four of the most significantly changed proteins in human monocytes were vimentin, myeloperoxidase, cytoskeleton-associated protein glycine-rich domain-containing linker protein 2, and zinc finger CCH domain-containing protein (7). According to these findings, thrombotic and obstetric APS may be two different subtypes. However, there are no biomarkers to differentiate TAPS from OAPS.

Urine proteomics has recently received increasing attention. Urine collection is an easy, non-invasive procedure. And it reflects the condition of the body in many aspects. It has been reported that urine proteins can serve as diagnostic biomarkers in rheumatic diseases. In systemic lupus erythematosus (SLE), urine proteins such as osteopontin N-half and urinary monocyte chemoattractant protein-1 could distinguish patients with lupus nephritis (LN) from those without LN (8, 9). Urinary cluster of differentiation was found to have a diagnostic value comparable to traditional serum biomarkers in rheumatoid arthritis (RA) (10). However, the role of urine proteomics in primary APS needs to be clarified. Therefore, we aimed to carry out urine proteomics in patients with primary APS to further identify TAPS from OAPS.

## Materials and Methods

### Patients

Primary APS patients (PAPS) were enrolled consecutively from 2016 to 2018 in Department of Rheumatology and Immunology in Ruijin Hospital, Shanghai. Patients who met the criteria for the classification of PAPS using the Sydney criteria were included (3). The diagnosis of PAPS was confirmed by two senior rheumatologists: Jialin Teng and Chengde Yang. Any APS patients secondary to other diseases such as SLE were excluded from this study. Finally, 15 patients with TAPS, 9 patients with OAPS, and 15 HCs were enrolled in the test cohort. To exclude obstetric complications, female patients in TAPS group were all with successful obstetric outcome. OAPS patients without the history of thrombosis were included. All HCs were recruited from age- matched volunteers with no history of autoimmune, rheumatic, or other diseases. All the patients recruited in our study were treatment-naïve, without receiving any anticoagulant therapy. Proteomics analysis was performed by protocol as previously used by our group to identify differentially expressed proteins in the urine (11). In the validation cohort, urine samples of 19 patients with primary OAPS, 21 patients with primary TAPS, 13 HCs with positive aPL antibodies (APL carriers), 20 patients with miscarriages (non-autoimmune), 21 patients with thrombosis (non-autoimmune), 30 patients with SLE, 30 patients with RA and 30 HCs were tested by enzyme-linked immunosorbent assay (ELISA). RA patients who met 2010 Rheumatoid Arthritis Classification Criteria were included (12). SLE patients were diagnosed using 2012 Systemic Lupus International Collaborating Clinics (SLICC) classification criteria (13). Patients with 3 unexplained miscarriages of less than 10 weeks were regarded as miscarriage, while patients with at least one unexplained death of a morphologically normal fetus of over 10 weeks were regarded as intrauterine death, excluding other causes. Thrombosis was defined according to the established criteria, using laboratory, imaging or Doppler, or histopathologic data. APL carriers were defined as HCs with positive aPL antibodies (either aCL, aβ2GPI or LAC). According to the Sydney criteria, positive aCL and aβ2GPI antibodies were defined as IgG and IgM aCL in the serum or plasma presented at a medium or high titer (more than 40 GPL or MPL, or the 99^th^ percentile), and IgG and IgM aβ2GPI at a titer over the 99^th^ percentile. These antibodies were measured by ELISA (Euroimmun, Germany) at least twice and 12 weeks apart. LAC was measured by the Automated Coagulation Laboratory 300R (Milan, Italy) according to the criteria of the International Society on Thrombosis and Haemostasis (ISTH) committee (14). All patients were screened using the dilute Russellgically normal fetus (dRVVT) testing and the activated partial thromboplastin time. The ratio of the dRVVT screening time/dRVVT confirming time over 1.20 was considered as LAC positive. Hypocomplementemia was defined as a low serum level of complement 3 and/or complement 4. Thrombocytopenia was defined as numbers of platelets less than 100*10^9^/L. Proteinuria was defined as more than 500mg/24h urine protein. Medical documents and laboratory tests were collected through the electronic system. Urine samples were collected early morning from inpatients excluding urinary tract infections, and frozen at -80°C until testing. The detection and analysis of these specimens were performed at the same time in the same batch in the test study and validation study, respectively. The study was performed under the Declaration of Helsinki and the Principles of Good Clinical Practice and approved by the Institutional Review Broad of Ruijin Hospital (ID:2016-62), Shanghai Jiao Tong University School of Medicine, Shanghai, China. Informed consent was obtained from the recruited subjects.

### Urine Sample Processing

Taking the relatively low concentration of the protein levels into account, every 5 patients’ urine samples were mixed into one, and analyzed SDT buffer (4% SDS, 100 mM DTT, 150 mM Tris-HCl, pH 8.0) was added to the sample and the lysate was boiled for 15 min. After centrifuged at 14000g for 40 min, the supernatant was quantified with the BCA Protein Assay Kit (Bio-Rad, USA). The proteins were separated on 12.5% SDS-PAGE gel. Protein bands were visualized by Coomassie Blue R-250 staining (Beyotime, Shanghai). 200 μg of proteins for each sample were incorporated into 30 μl SDT buffer. The detergent, DTT and other low-molecular-weight components were removed using UA buffer, which was 8 M Urea, 150 mM Tris-HCl pH 8.0, by repeated ultrafiltration (Microcon units, 10 kD, Germany). Then 100 μl iodoacetamide (100 mM IAA in UA buffer) was added to block reduced cysteine residues and the samples were incubated for 30 min in darkness. The filters were washed with 100 μl UA buffer three times and then 100 μl Dissolution buffer (DS buffer) twice. Finally, the protein suspensions were digested with 4 μg trypsin (Promega, USA) in 40 μl DS buffer overnight at 37°C, and the resulting peptides were collected as a filtrate. The peptides of each sample were desalted on C18 Cartridges (Empore™ SPE Cartridges C18 (standard density), bed I.D. 7 mm, volume 3 ml, Sigma, USA), concentrated by vacuum centrifugation and reconstituted in 40 µl of 0.1% (v/v) formic acid.

### iTRAQ Labeling

100 μg peptide mixture of each sample was labeled using iTRAQ reagent according to the manufacturer’s instructions (Applied Biosystems, USA). ITRAQ labeled peptides were fractionated by Strong Cation Exchange (SCX) chromatography using the AKTA Purifier system (GE Healthcare, USA). The dried peptide mixture was reconstituted and acidified with buffer A (10 mM KH_2_PO4 in 25% of ACN, pH 3.0) and loaded onto a PolySULFOETHYL 4.6 x 100 mm column (5 µm, 200 Å, PolyLC Inc, Maryland, USA). The peptides were eluted at a flow rate of 1 ml/min with a gradient of 0%–8% buffer B (500 mM KCl, 10 mM KH_2_PO4 in 25% of ACN, pH 3.0) for 22 min, 8–52% buffer B during 22-47 min, 52%-100% buffer B during 47-50 min, 100% buffer B during 50-58 min, and buffer B was reset to 0% after 58min. The elution was monitored by absorbance at 214 nm, and fractions were collected every 1 min. The collected fractions were desalted on C18 Cartridges (Empore™ SPE Cartridges C18 (standard density), bed I.D. 7 mm, volume 3 ml, Sigma), and concentrated by vacuum centrifugation.

### LC-MS/MS Analysis

LC-MS/MS analysis was performed on a Q Exactive mass spectrometer (Thermo Scientific, USA) that was coupled to Easy nLC (Proxeon Biosystems, now Thermo Fisher Scientific, USA) for 60 min (determined by project proposal). The mass spectrometer was operated in positive ion mode. MS data were acquired using a data-dependent top10 method dynamically choosing the most abundant precursor ions from the survey scan (350-1800 m/z) for (high energy collision dissociation (HCD) fragmentation. The automatic gain control (AGC) target was set to 3e6, and maximum inject time to 50 ms. Survey scans were acquired at a resolution of 70,000 at m/z 200 and resolution for HCD spectra was set to 17,500 at m/z 200, and isolation width was 2 m/z. The normalized collision energy was 30 eV. All peptide ratios were normalized by the median protein ratio. The median protein ratio should be 1 after the normalization.

### Enzyme-Linked Immunosorbent Assay

The urine concentration of C-X-C motif chemokine ligand 12 (CXCL12 or stromal cell-derived factor 1, SDF-1), platelet-derived growth factor subunit B (PDGFB), matrix metalloproteinase3 (MMP3), and platelet-derived growth factor receptor alpha (PDGFRA) were measured by human ELISA assay kit (R&D, USA). ELISA was performed according to the manufacture’s instruction.

### Statistical Analysis

Blast2GO was used in gene ontology (GO) enrichment analysis to annotate the target protein set, and the bar graph was drawn. Cluster 3.0 was used in cluster analysis. R language pack was used to make the Venn diagram and volcano plots. All data were statistically analyzed using the Statistical Package for the Social Sciences for Windows (V.23.0; SPSS, IBM). Graphs were drawn using GraphPad Prism software 8.0 (GraphPad Software, USA). Quantitative data between two groups with a Gaussian distribution were analyzed using an unpaired *t*-test. ANOVA was performed to compare the differences among multiple groups. *Chi*-square test and Kruskal-Wallis test were carried out for comparisons of two or more than two groups with categorical variable, respectively. Spearman rank-order correlation analysis was performed to calculate the correlation coefficient and *p* value between specific protein and aPLs. Receiver operating characteristic (ROC) curves and the area under the ROC curve (AUC) were used to access the sensitivity and specificity. In the test study, fold change of more than 1.2 or less than 0.83 was considered significant. *P*<0.05 was considered statistically significant.

## Results

### Clinical Characteristics of Patients With APS

The clinical characteristics of patients with primary APS and HCs enrolled in the test cohort were presented in **Table 1**. Fifteen patients with TAPS, 9 patients with OAPS, and 15 HCs were included. Then, 19 patients with primary OAPS, 21 patients with primary TAPS, 13 APL carriers, 20 patients with miscarriages (non-autoimmune), 21 patients with thrombosis (non-autoimmune), 30 patients with SLE, 30 patients with RA, and 30 HCs were included in the validation cohort, and the clinical data were shown in **Table 2**.

**Table 1:** Clinical and laboratory features of patients with TAPS, OAPS and HCs in the iTRAQ study.

	TAPS(n=15)	OAPS (n=9)	HCs (n=15)	*P* value
Age (mean ± SD)	41.1 ± 15.3	34.8 ± 5.0	42.1 ± 14.1	0.402
Sex (female/male)	10/5	9/0	13/2	0.054
Duration (months)	2.1 ± 2.1	4.3 ± 3.7	/	0.125
Venous thrombosis	15 (100.0%)	0 (0.0%)	/	<0.001
Arterial thrombosis	5 (33.3%)	0 (0.0%)	/	0.118
Miscarriage, <10 weeks	0 (0.0%)	6 (66.7%)	/	0.001
Intrauterine death, >10 weeks	0 (0.0%)	4 (44.4%)	/	0.012
LAC positive	10 (66.7%)	6 (66.7%)	/	1.000
IgG aCL positive	4 (26.7%)	3 (33.3%)	/	1.000
IgM aCL positive	2 (13.3%)	1 (11.1%)	/	1.000
IgG aβ2GPI positive	4 (26.7%)	3 (33.3%)	/	1.000
IgM aβ2GPI positive	1 (6.7%)	3 (33.3%)	/	0.130
ANA positive	0 (0.0%)	0 (0.0%)	/	/
Anti-dsDNA positive	1 (6.7%)	1 (11.1%)	/	1.000
Hypocomplementemia	3 (20.0%)	4 (44.4%)	/	0.356
Thrombocytopenia	7 (46.7%)	3 (33.3%)	/	0.678
ESR (mm/h)	38.2 ± 42.5	19.1 ± 12.5	/	0.233
aGAPSS	8.3 ± 4.2	9.7 ± 3.9	/	0.447
Proteinuria	1 (6.7%)	1 (11.1%)	/	1.000

iTRAQ, isobaric tags for relative and absolute quantification; TAPS, thrombotic antiphospholipid syndrome; OAPS, obstetric antiphospholipid syndrome; HCs, healthy controls; SD, standard deviation; LAC, lupus anticoagulant; aCL, antibodies to cardiolipin; aβ2GPI, antibodies to β2-glycoprotein I; ESR, erythrocyte sedimentation rate; aGAPSS, adjusted Global Antiphospholipid Syndrome Score.

**Table 2:** Clinical and laboratory features of patients with TAPS, patients with OAPS, APL carriers, patients with miscarriages, patients with thrombosis, SLE, RA and HCs in the validation cohort.

	TAPS (n=21)	OAPS (n=19)	APL carriers (n=13)	Miscarriages (n=20)	Thrombosis (n=21)	SLE (n=30)	RA (n=30)	HCs (n=30)	*P* value
Age (Mean ± SD)	40.9 ± 11.1	35.6 ± 8.6	38.4 ± 16.0	32.0 ± 4.7	40.2 ± 12.1	31.6 ± 5.1	36.4 ± 14.5	35.5 ± 8.2	0.063
Sex (female/male)	13/8	19/0	11/2	20/0	13/8	27/3	25/5	19/11	0.001
Duration(months)	3.8 ± 6.5	4.7 ± 6.8	/	/	/	2.8 ± 2.6	2.7 ± 2.2	/	0.654
Venous thrombosis	21 (100.0%)	0 (0.0%)	0 (0.0%)	0 (0.0%)	9 (42.9%)	/	/	/	<0.001
Arterial thrombosis	6 (28.6%)	0 (0.0%)	0 (0.0%)	0 (0.0%)	12 (57.1%)	/	/	/	<0.001
Miscarriage, <10 weeks	0 (0.0%)	9 (47.4%)	0 (0.0%)	20 (100.0%)	/	/	/	/	<0.001
Intrauterine death, >10 weeks	0 (0.0%)	14 (73.7%)	0 (0.0%)	0 (0.0%)	/	/	/	/	<0.001
LAC positive	12 (57.1%)	15 (78.9%)	9 (69.2%)	/	/	/	/	/	0.341
IgG aCL positive	7 (33.3%)	8 (42.1%)	5 (38.5%)	/	/	/	/	/	0.850
IgM aCL positive	2 (9.5%)	1 (5.3%)	5 (38.5%)	/	/	/	/	/	0.025
IgG aβ2GPI positive	4 (19.0%)	6 (31.6%)	2 (15.4%)	/	/	/	/	/	0.500
IgM aβ2GPI positive	1 (4.7%)	4 (21.1%)	0 (0.0%)	/	/	/	/	/	0.091
ANA positive	0 (0.0%)	0 (0.0%)	/	/	/	30 (100.0%)	0 (0.0%)	/	<0.001
Anti-dsDNA positive	3 (14.3%)	3 (15.8%)	/	/	/	29 (96.7%)	0 (0.0%)	/	<0.001
Hypocomplementemia	6 (28.6%)	8 (42.1%)	/	/	/	25 (83.3%)	/	/	<0.001
Thrombocytopenia	10 (47.6%)	5 (26.3%)	/	/	/	8 (26.7%)	/	/	0.227
ESR (mm/h)	30.9 ± 37.9	24.0 ± 19.8	/	/	/	60.2 ± 37.9	20.6 ± 17.5	/	<0.001
aGAPSS	8.7 ± 3.9	9.8 ± 3.7	/	/	/	/	/	/	0.356
Proteinuria	2 (9.5%)	1 (5.3%)	/	/	/	14 (46.7%)	0 (0.0%)	/	<0.001

TAPS, thrombotic antiphospholipid syndrome; OAPS, obstetric antiphospholipid syndrome; APL, antiphospholipid antibody; SD, standard deviation; LAC, lupus anticoagulant; aCL, antibodies to cardiolipin; aβ2GP1, antibodies to β2-glycoprotein I; ESR, erythrocyte sedimentation rate; aGAPSS, adjusted Global Antiphospholipid Syndrome Score; SLE, systemic lupus erythematosus; RA, rheumatoid arthritis; HCs, healthy controls.

### Comparison of Patients With TAPS, Patients With OAPS and HCs in the iTRAQ Study

**Figure 1:**
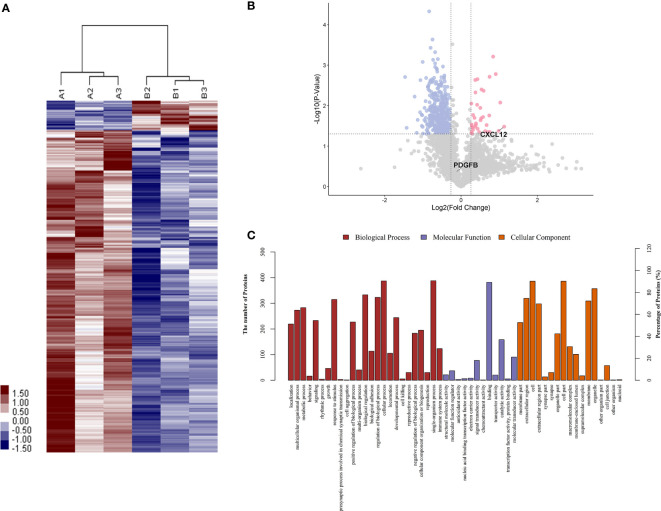
The differentiated urine proteins between patients with TAPS and HCs analyzed by iTRAQ study. **(A)** Cluster analysis of patients with TAPS with HCs (A represented HCs; B represented TAPS). **(B)** Volcano plot showed that CXCL12 was in the upper right quadrant, which was in pink color area. **(C)** Gene ontology (GO) analysis of TAPS *vs* HCs. iTRAQ, isobaric tags for relative and absolute quantification; TAPS, thrombotic antiphospholipid syndrome; HCs, healthy controls.

**Figure 2:**
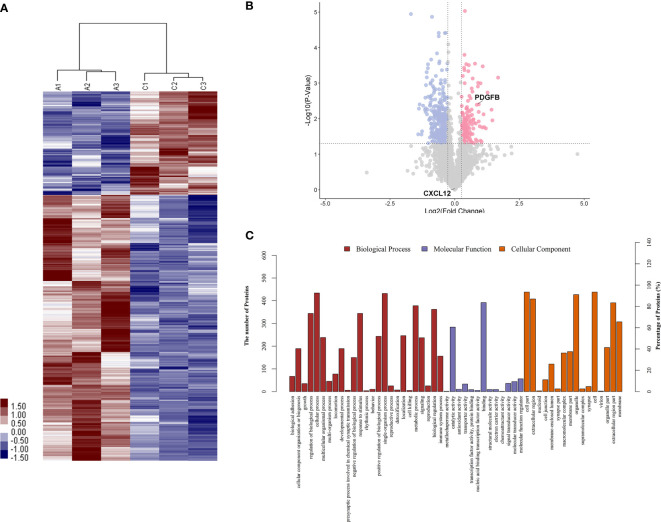
The differentiated urine proteins between patients with OAPS and HCs analyzed by iTRAQ study. **(A)** Cluster analysis of patients with OAPS with HCs (A represented HCs; C represented OAPS). **(B)** Volcano plot showed that PDGFB was in the upper right quadrant, which was in pink color area. **(C)** Gene ontology (GO) analysis of OAPS *vs* HCs. iTRAQ, isobaric tags for relative and absolute quantification; OAPS, obstetric antiphospholipid syndrome; HCs, healthy controls.

**Figure 3:**
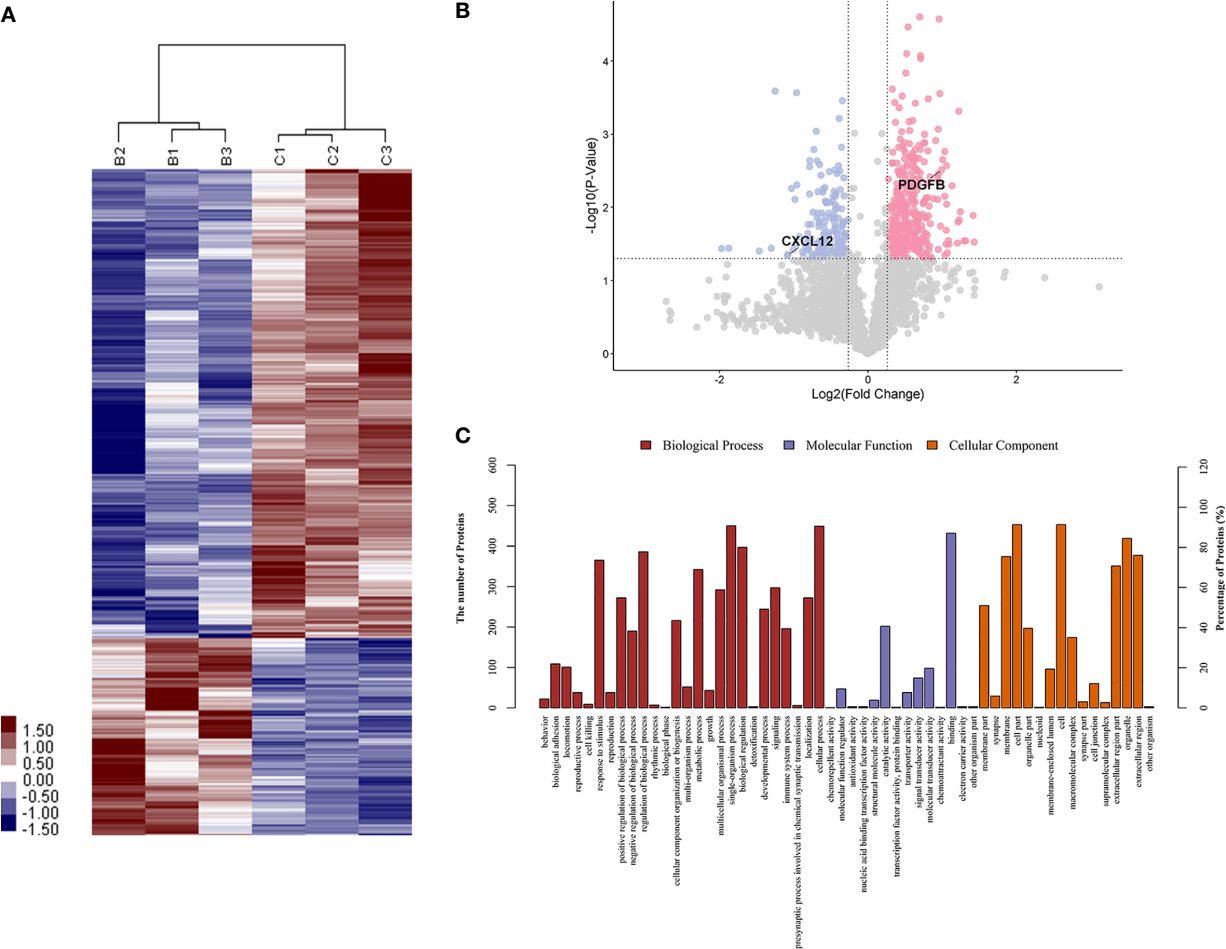
The differentiated urine proteins between patients with OAPS and patients with TAPS analyzed by iTRAQ study. **(A)** Cluster analysis of OAPS with TAPS (B represented TAPS; C represented OAPS). **(B)** Volcano plot showed that PDGFB was in the upper right quadrant, which was in pink color area. CXCL12 was in the upper left quadrant, which was in blue color area. **(C)** Gene ontology (GO) analysis of OAPS *vs* TAPS. iTRAQ, isobaric tags for relative and absolute quantification; OAPS, obstetric antiphospholipid syndrome; HCs, healthy controls.

To identify proteins that were differentially expressed in primary APS (TAPS and OAPS) and HCs, iTRAQ was performed on the urine in these samples. Data interpretation was divided into three groups: TAPS *vs*. HCs, OAPS *vs*. HCs, and OAPS *vs*. TAPS. Cluster analysis (**Figures 1**, **2** and **3A**) demonstrated that there were 36 upregulated proteins and 392 downregulated proteins, which added up to a total of 428 proteins in TAPS *vs*. HCs. In addition, there were 132 upregulated proteins and 337 downregulated proteins, which summed up to 469 proteins in OAPS *vs*. HCs. Furthermore, there were 349 upregulated proteins and 147 downregulated proteins, which added up to 496 proteins in OAPS *vs*. TAPS (**Supplementary Table 1**). Moreover, in the Venn diagram (**Supplementary Figure 1**), a total of 136 proteins changed in TAPS *vs*. HCs, 236 proteins in OAPS *vs*. HCs, and 211 proteins in OAPS *vs*. TAPS. Proteins with fold change values >1.2 or <0.83, and *p* values <0.05, were selected for further screening. The 10 most secreted urine proteins were identified as potential biomarkers among the TAPS, OAPS, and HC groups (**Supplementary Tables 2–4**). GO analysis comprised three parts: biological processes, molecular functions, and cellular components (**Figures 1** **–** **3C**). It indicated that cytokines and cytokine receptors were present, as reported in APS, which might play an important role (15). Then, CXCL12, PDGFB, MMP3 and PDGFRA screened out in the iTRAQ study were validated by ELISA in the validation cohort.

### Confirmation of CXCL12 and PDGFB in the Urine of Patients With TAPS and OAPS by ELISA in the Validation Cohort

**Figure 4:**
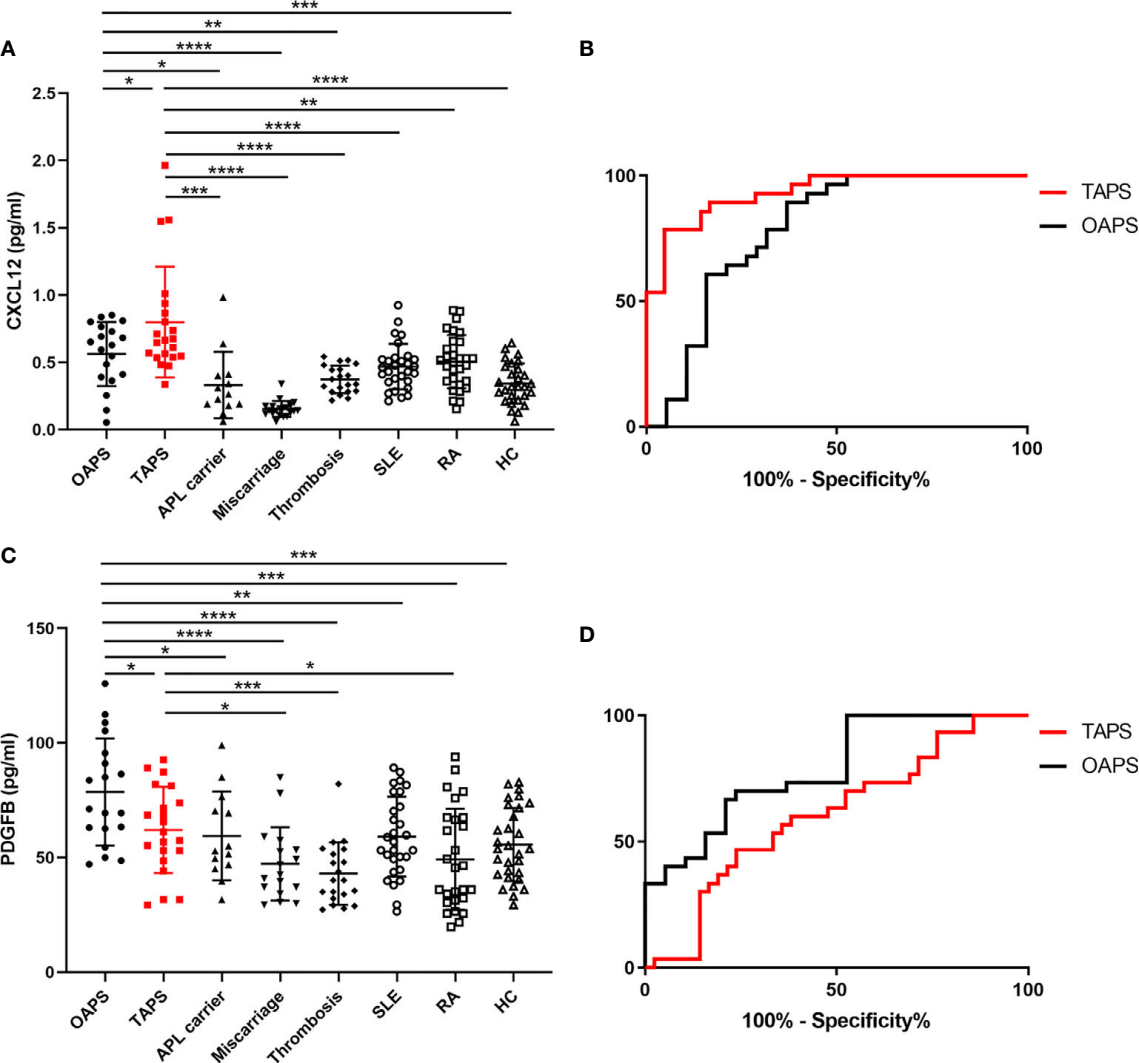
Validation of CXCL12 and PDGFB as urinary biomarkers for patients with OAPS and patients with TAPS, respectively. **(A)** CXCL12 levels in the urine of patients with OAPS, patients with TAPS, APL carriers, patients with miscarriages, patients with thrombosis, systemic lupus erythematosus (SLE), rheumatoid arthritis (RA) and healthy controls (HCs). **(B)** The ROC curves of CXCL12 levels in the urine of TAPS *vs* HCs and that of OAPS *vs* HCs. **(C)** PDGFB levels in the urine of OAPS, TAPS, APL carriers, patients with miscarriages, patients with thrombosis, SLE, RA and HCs. **(D)** The ROC curves of PDGFB levels in the urine of TAPS *vs* HCs and that of OAPS *vs* HCs. *p < 0.05, **p < 0.01, ***p < 0.001, ****p < 0.0001.

In the validation study, urine samples from 19 patients with primary OAPS, 21 patients with primary TAPS, 13 APL carriers, 20 patients with miscarriages (non-autoimmune), 21 patients with thrombosis (non-autoimmune), 30 patients with RA, 30 patients with SLE, and 30 HCs were tested by ELISA. As shown in **Figure 4**, CXCL12 levels were higher in the urine of patients with TAPS than in those with OAPS (*p*=0.0350), APL carriers (*p*=0.0008), patients with miscarriages (*p*<0.0001), patients with thrombosis (*p*<0.0001), patients with RA (*p*=0.0012), patients with SLE (*p*<0.0001), and HCs (*p*<0.0001). CXCL12 levels were higher in patients with OAPS than in APL carriers (*p*=0.0123), patients with miscarriages (*p*<0.0001), patients with thrombosis (*p*=0.0024), and HCs (*p*=0.0007), but showed no differences compared to patients with RA (*p*=0.2510) or SLE (*p*=0.0791). On the other hand, PDGFB levels were higher in the urine of patients with OAPS than in those with TAPS (*p*=0.0406), APL carriers (*p*=0.0207), patients with miscarriages (*p*<0.0001), patients with thrombosis (*p*<0.0001), patients with RA (*p*=0.0002), patients with SLE (*p*=0.0052), and HCs (*p*=0.0005). Urinary PDGFB levels were higher in patients with TAPS than in those with RA (*p*=0.0425), with miscarriages (*p*=0.0144) and with thrombosis (*p*=0.0007). In addition, PDGFB levels in patients with TAPS was not significantly different from SLE (*p*=0.5294) or HCs (*p*=0.2067). In ROC curves, TAPS *vs*. HCs showed a higher AUC of 0.9328 (95% confidence interval [CI]: 0.8670-0.9987) (*p*<0.0001) than that of OAPS *vs*. HCs, which was 0.7867 (95% CI: 0.6375-0.9358) (*p*=0.0009) in urinary CXCL12 levels. In terms of urinary PDGFB levels, the AUC of OAPS *vs*. HCs was higher (AUC: 0.7886, 95% CI: 0.6594-0.9178) (*p*=0.0007) than that of TAPS *vs*. HCs (AUC: 0.6056, 95% CI: 0.4434-0.7678) (*p*=0.2031). In addition, there were no differences in the MMP3 (*p*=0.449) and PDGFRA (*p*=0.217) levels between OAPS and TAPS (**Supplementary Figure 2**).

### Comparison of CXCL12, PDGFB and Clinical Features in the Validation Cohort

**Figure 5:**
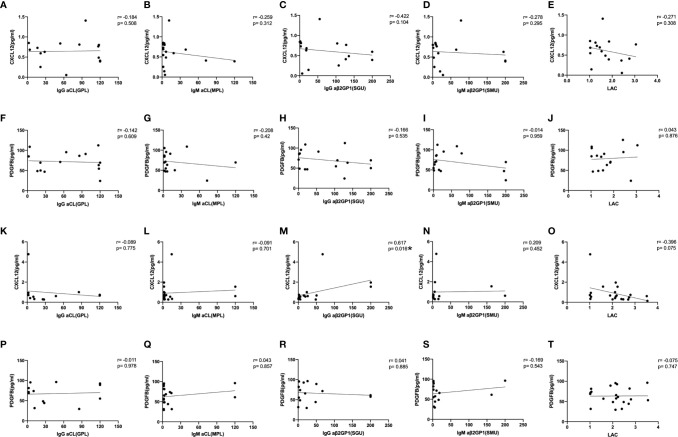
The correlation between the levels of CXCL12, PDGFB with aPLs. The spearman rank-order correlation between the levels of CXCL12, PDGFB and anti-cardiolipin antibody (aCL), anti-β2-glycoprotein I antibody (aβ2GPI) and lupus anticoagulant (LAC) in OAPS patients **(A–J)** and TAPS patients **(K–T)**. *p < 0.05.

Both the iTRAQ study and validation test indicated the specificity of CXCL12 and PDGFB in differentiating OAPS from TAPS. To better understand the relationship between CXCL12, PDGFB, and the two APS subgroups, we further analyzed the correlation between the CXCL12, PDGFB, and aPL levels with different clinical characteristics. First, Spearman rank-order correlation analysis was used to evaluate the correlation between the levels of CXCL12, PDGFB, and aPLs (aCL, aβ2GPI, and LAC), respectively. It showed that CXCL12 levels were positively correlated with IgG aβ2GPI antibody (*r*=0.617, *p*=0.016) (**Figure 5**). In addition, there were no differences between the CXCL12 and PDGFB levels in patients with OAPS and TAPS with single, double, triple positive aPLs (**Supplementary Figure 3**). In patients with OAPS, the CXCL12 and PDGFB levels were analyzed according to the number of adverse pregnancy outcomes, and no differences were observed (**Supplementary Figure 4**). Moreover, in TAPS, the CXCL12 and PDGFB levels were also analyzed according to the number of thromboses and the presence or absence of arterial thrombosis, which indicated that there were no differences (**Supplementary Figure 5**).

## Discussion

Urine biomarkers have been widely discussed in recent years. It has been reported that there were more than 2300 proteins in the urine (16). It has also been reported that neutrophil gelatinase-associated lipocalin (NGAL), high mobility group box-1 (HMGB-1), vascular cell adhesion molecule-1 (VCAM-1), vitamin D-binding protein (VDBP) levels showed diagnostic performance for discriminating patients with LN from those without LN (17–20). Besides, collecting urine samples is more convenient and causes less pain in patients than while collecting blood samples. We previously reported a study in adult-onset Still’s disease (AOSD) and found that urine α-1-acid glycoprotein 1 (LRG1), orosomucoid 1 (ORM1), and ORM2 might be new biomarkers of AOSD (11). Similarly, in this study, we analyzed urine proteomics to identify new biomarkers that might help differentiate TAPS from OAPS.

We used iTRAQ to screen for different proteins in the urine of patients with TAPS, patients with OAPS, and HCs. The results demonstrated that TAPS *vs*. HCs, OAPS *vs*. HCs, and OAPS *vs*. TAPS exhibited varied panels of upregulated and downregulated proteins, in which the CXCL12 levels were higher in the urine of those with TAPS than OAPS, while the PDGFB levels were lower in the urine of those with TAPS than OAPS. Then, CXCL12 and PDGFB levels were validated by ELISA in the validation cohort. Besides, we analyzed the correlation between CXCL12, PDGFB, and aPLs. The CXCL12 levels were positively correlated with IgG aβ2GPI antibody. Many previous studies manifested that cytokine played an important role in autoimmune diseases (21, 22). Thus, we investigated this aspect and assumed that CXCL12 and PDGFB might have the potential to differentiate TAPS from OAPS.

CXCL12 has been reported as stromal cell-derived factor-1 (SDF-1), a chemokine that plays an important role in the regulation of migration, proliferation, and differentiation of hematopoietic cells (23). CXCL12 fulfills its functions in homeostatic and pathological conditions by interacting with its receptors C-X-C chemokine receptor 4 (CXCR4) and atypical chemokine receptor 3 (ACKR3). Imbalances in the CXCL12/CXCR4/ACKR3 axis are associated with diseases, including cancer, multiple sclerosis, and RA (23). In addition, it has been reported that plasma CXCL12 levels were significantly elevated in the blood of patients with TAPS regardless of the arterial/venous nature of the thrombosis compared with HCs (24), which was similar to the results of our study. CXCL12 increases the chemotaxis of inflammatory cells and contributes to the activation of platelets in the damaged area, which could result in thrombosis or acceleration of the damage to the vascular integrity (25, 26). These studies demonstrated that CXCL12 might play an important role in the pathogenesis of TAPS. As reported previously, the functional role of the CXCL12 801 genotype involves the upregulation of CXCL12 protein. In a genomic study, patients with SLE with and without APS demonstrated different distributions of CXCL12 G801A genotype frequencies. SLE patients with APS demonstrated an increased frequency of the CXCL12 A allele and AA genotype compared with patients without APS, suggesting the clinical relevance of this polymorphism (27). It is promising that blockade of chemokine ligand 2 and CXCL12 could be as effective as cyclophosphamide in suppressing proliferative LN (28). It has also been indicated that the CXCR4-CXCL12 axis could be regarded as a potential therapeutic target because of its importance for antibody-secreting cells’ homing and survival in lupus-prone mice (29).

PDGFB is mainly expressed in vascular endothelial cells, megakaryocytes, and neurons. PDGFB produced by endothelial cells drives the proliferation and spreading of vascular smooth muscle cells and pericytes in conjunction with angiogenesis (30). In addition, PDGFR-β and PDGFB appear to play a role in neuronal cardiac neural crest development, as both PDGFR-β and PDGFB knockout mice displayed abnormal cardiac innervation (30). Moreover, it has been reported that the toll-like receptor 9 (TLR9)/transforming growth factor-β1 (TGF-β1)/PDGFB pathway can be activated in SLE. Elevated PDGFB might contribute to the proliferation of renal mesangial cells and may be involved in the development of LN (31). However, more studies are needed to clarify the role of PDGFB in patients with APS.

Apart from these two cytokines, other biomarkers of APS have also been reported. Surface-enhanced laser desorption/ionization-time of flight analysis made it possible to discriminate between several proteins in women with pregnancy morbidity with and without aPLs, in which nine proteins were found in significantly higher levels in aPL-positive women (32). Apolipoprotein H and mitogen-activated protein kinase, previously described in the pathogenesis of APS, were found to differ in APS from non-APS patients with thrombosis (33). Patients with OAPS may also develop thrombosis. A total of 63% of women with OAPS developed thrombosis after initial obstetric morbidity. Women with subsequent thrombosis after OAPS had a higher adjusted global APS score (aGAPSS) (34).

There are several limitations to this study. First, the number of patients with primary APS was small. We included treatment-naïve patients with primary APS, which added the difficulty of recruiting patients. A larger group of patients are needed to verify the results. Second, not all patients in the TAPS group were females. There were no differences in the urinary CXCL12 and PDGFB levels between male and female in patients with TAPS (*p*>0.05) (**Supplementary Figure 6**). Selecting only female patients with TAPS for this study might cause selection bias. Therefore, we did not exclude male patients with TAPS.

In conclusion, the iTRAQ analysis showed that TAPS and OAPS distributed different urine protein patterns. Results of the validation study indicated that urinary CXCL12 and PDGFB might serve as potential biomarkers to differentiate primary TAPS from primary OAPS.

## Data Availability Statement

The data presented in the study are deposited in the iProX repository, accession number: IPX0003233000.

## Ethics Statement

The studies involving human participants were reviewed and approved by the Institutional Review Broad of Ruijin Hospital. The patients/participants provided their written informed consent to participate in this study.

## Author Contributions

CS, CY and JY conceived of the study and participated in its design and coordination. ZZ, YY and FW carried out the ELISA. YS, JT, HL, XC, YTS, HS, LW, TL, MW collected samples and contributed to data acquisition, analysis, and critical review for intellectual content. QH, HC and JJ performed the statistical analyses for all the data. ZZ, JY, YY, and FW drafted the manuscript and revised the manuscript. All authors contributed to the article and approved the submitted version.

## Funding

This work was supported by National Natural Science Foundation of China (81801592), Clinical Research Plan of SHDC (SHDC2020CR4011), Ruijin Hospital Youth Incubation Project (KY2021607) and Shanghai Pujiang Young Rheumatologists Training Program (SPROG202006).

## Conflict of Interest

The authors declare that the research was conducted in the absence of any commercial or financial relationships that could be construed as a potential conflict of interest.

## Publisher’s Note

All claims expressed in this article are solely those of the authors and do not necessarily represent those of their affiliated organizations, or those of the publisher, the editors and the reviewers. Any product that may be evaluated in this article, or claim that may be made by its manufacturer, is not guaranteed or endorsed by the publisher.

## References

[1] Giannakopoulos B Passam F Ioannou Y Krilis SA. How We Diagnose the Antiphospholipid Syndrome. Blood (2009) 113:985–94. 10.1182/blood-2007-12-129627 18755986

[2] Galli M Barbui T. Antiprothrombin Antibodies: Detection and Clinical Significance in the Antiphospholipid Syndrome. Blood (1999) 93:2149–57. 10.1182/blood.V93.7.2149 10090921

[3] Miyakis S Lockshin MD Atsumi T Branch DW Brey RL Cervera R. International Consensus Statement on an Update of the Classification Criteria for Definite Antiphospholipid Syndrome (APS). J Thromb Haemost (2006) 4:295–306. 10.1111/j.1538-7836.2006.01753.x 16420554

[4] Tektonidou MG Andreoli L Limper M Tincani A Ward MM. Management of Thrombotic and Obstetric Antiphospholipid Syndrome: A Systematic Literature Review Informing the EULAR Recommendations for the Management of Antiphospholipid Syndrome in Adults. RMD Open (2019) 5:e000924. 10.1136/rmdopen-2019-000924 31168416 PMC6525610

[5] Lopez-Pedrera C Barbarroja N Aguirre MA Torres LA Velasco F Cuadrado MJ. Genomics and Proteomics: A New Approach for Assessing Thrombotic Risk in Autoimmune Diseases. Lupus (2008) 17:904–15. 10.1177/0961203308095285 18827055

[6] Ripoll VM Pregnolato F Mazza S Bodio C Grossi C McDonnell T. Gene Expression Profiling Identifies Distinct Molecular Signatures in Thrombotic and Obstetric Antiphospholipid Syndrome. J Autoimmun (2018) 93:114–23. 10.1016/j.jaut.2018.07.002 PMC6123515 30033000

[7] Ripoll VM Lambrianides A Pierangeli SS Poulton K Ioannou Y Heywood WE. Changes in Regulation of Human Monocyte Proteins in Response to IgG From Patients With Antiphospholipid Syndrome. Blood (2014) 124:3808–16. 10.1182/blood-2014-05-577569 PMC4263988 25301710

[8] Kitagori K Yoshifuji H Oku T Sasaki C Miyata H Mori KP. Cleaved Form of Osteopontin in Urine as a Clinical Marker of Lupus Nephritis. PloS One (2016) 11:e0167141. 10.1371/journal.pone.0167141 27992535 PMC5167225

[9] Gupta R Yadav A Aggarwal A. Longitudinal Assessment of Monocyte Chemoattractant Protein-1 in Lupus Nephritis as a Biomarker of Disease Activity. Clin Rheumatol (2016) 35:2707–14. 10.1007/s10067-016-3404-9 27624649

[10] Kang MJ Park YJ You S Yoo SA Choi S Kim DH. Urinary Proteome Profile Predictive of Disease Activity in Rheumatoid Arthritis. J Proteome Res (2014) 13:5206–17. 10.1021/pr500467d 25222917

[11] Sun Y Wang F Zhou Z Teng J Su Y Chi H. Urinary Proteomics Identifying Novel Biomarkers for the Diagnosis of Adult-Onset Still’s Disease. Front Immunol (2020) 11:2112. 10.3389/fimmu.2020.02112 33013889 PMC7500098

[12] Aletaha D Neogi T Silman AJ Funovits J Felson DT Bingham CO. 2010 Rheumatoid Arthritis Classification Criteria: An American College of Rheumatology/European League Against Rheumatism Collaborative Initiative. Ann Rheum Dis (2010) 69:1580–8. 10.1136/ard.2010.138461 20699241

[13] Petri M Orbai AM Alarcon GS Gordon C Merrill JT Fortin PR. Derivation and Validation of the Systemic Lupus International Collaborating Clinics Classification Criteria for Systemic Lupus Erythematosus. Arthritis Rheum (2012) 64:2677–86. 10.1002/art.34473 PMC3409311 22553077

[14] Yelnik CM Laskin CA Porter TF Branch DW Buyon JP Guerra MM. Lupus Anticoagulant is the Main Predictor of Adverse Pregnancy Outcomes in aPL-Positive Patients: Validation of PROMISSE Study Results. Lupus Sci Med (2016) 3:e000131. 10.1136/lupus-2015-000131 26835148 PMC4716418

[15] Petri M. Antiphospholipid Syndrome. Transl Res (2020) 225:70–81. 10.1016/j.trsl.2020.04.006 32413497 PMC7487027

[16] Kentsis A Monigatti F Dorff K Campagne F Bachur R Steen H. Urine Proteomics for Profiling of Human Disease Using High Accuracy Mass Spectrometry. Proteomics Clin Appl (2009) 3(9):1052–61. 10.1002/prca.200900008 PMC2994589 21127740

[17] Soliman S Mohamed FA Ismail FM Stanley S Saxena R Mohan C. Urine Angiostatin and VCAM-1 Surpass Conventional Metrics in Predicting Elevated Renal Pathology Activity Indices in Lupus Nephritis. Int J Rheum Dis (2017) 20:1714–27. 10.1111/1756-185X.13197 29076253

[18] Go DJ Lee JY Kang MJ Lee EY Lee EB Yi EC. Urinary Vitamin D-Binding Protein, A Novel Biomarker for Lupus Nephritis, Predicts the Development of Proteinuric Flare. Lupus (2018) 27:1600–15. 10.1177/0961203318778774 29958502

[19] El Shahawy MS Hemida MH Abdel-Hafez HA El-Baz TZ Lotfy AM Emran TM. Urinary Neutrophil Gelatinase-Associated Lipocalin as a Marker for Disease Activity in Lupus Nephritis. Scand J Clin Lab Invest (2018) 78(4):264–8. 10.1080/00365513.2018.1449242 29533691

[20] Jog NR Blanco I Lee I Putterman C Caricchio R. Urinary High-Mobility Group Box-1 Associates Specifically With Lupus Nephritis Class V. Lupus (2016) 25(14):1551–7. 10.1177/0961203316644331 PMC5061582 27075010

[21] Pacheco Y Barahona-Correa J Monsalve DM Acosta-Ampudia Y Rojas M Rodriguez Y. Cytokine and Autoantibody Clusters Interaction in Systemic Lupus Erythematosus. J Transl Med (2017) 15:239. 10.1186/s12967-017-1345-y 29178890 PMC5702157

[22] Davis LS Reimold AM. Research and Therapeutics-Traditional and Emerging Therapies in Systemic Lupus Erythematosus. Rheumatol (Oxford) (2017) 56:i100–13. 10.1093/rheumatology/kew417 PMC5850311 28375452

[23] Janssens R Struyf S Proost P. The Unique Structural and Functional Features of CXCL12. Cell Mol Immunol (2018) 15(4):299–311. 10.1038/cmi.2017.107 29082918 PMC6052832

[24] Smadja D Gaussem P Roncal C Fischer AM Emmerich J Darnige L. Arterial and Venous Thrombosis Is Associated With Different Angiogenic Cytokine Patterns in Patients With Antiphospholipid Syndrome. Lupus (2010) 19:837–43. 10.1177/0961203309360985 20133349

[25] Gerli G VC Turri O Erario M Gardellini A Pugliano M Biondi ML. SDF1-3’A Gene Polymorphism Is Associated With Chronic Myeloproliferative Disease and Thrombotic Events. Clin Chem (2005) 51(12):2411–4. 10.1373/clinchem.2005.057802 16306115

[26] Weber C Schober A Zernecke A. Chemokines: Key Regulators of Mononuclear Cell Recruitment in Atherosclerotic Vascular Disease. Arterioscler Thromb Vasc Biol (2004) 24(11):1997–2008. 10.1161/01.ATV.0000142812.03840.6f 15319268

[27] Lima G Soto-Vega E Atisha-Fregoso Y Sanchez-Guerrero J Vallejo M Vargas-Alarcon G. MCP-1, RANTES, and SDF-1 Polymorphisms in Mexican Patients With Systemic Lupus Erythematosus. Hum Immunol (2007) 68:980–5. 10.1016/j.humimm.2007.10.007 18191726

[28] Devarapu SK Kumar Vr S Rupanagudi KV Kulkarni OP Eulberg D Klussmann S. Dual Blockade of the Pro-Inflammatory Chemokine CCL2 and the Homeostatic Chemokine CXCL12 Is as Effective as High Dose Cyclophosphamide in Murine Proliferative Lupus Nephritis. Clin Immunol (2016) 169:139–47. 10.1016/j.clim.2016.07.003 27392463

[29] Cheng Q Khodadadi L Taddeo A Klotsche J FH B Radbruch A. CXCR4-CXCL12 Interaction Is Important for Plasma Cell Homing and Survival in NZB/W Mice. Eur J Immunol (2018) 48:1020–9. 10.1002/eji.201747023 29427452

[30] Andrae J Gallini R Betsholtz C. Role of Platelet-Derived Growth Factors in Physiology and Medicine. Genes Dev (2008) 22(10):1276–312. 10.1101/gad.1653708 PMC2732412 18483217

[31] Yuan Y Yang M Wang K Sun J Song L Diao X. Excessive Activation of the TLR9/TGF-Beta1/PDGF-B Pathway in the Peripheral Blood of Patients With Systemic Lupus Erythematosus. Arthritis Res Ther (2017) 19:70. 10.1186/s13075-017-1238-8 28356164 PMC5372299

[32] Alvarez AM Neubeck S Parra S Markert UR Cadavid AP. Serum Protein Profile in Women With Pregnancy Morbidity Associated With Antiphospholipid Syndrome. J Hum Reprod Sci (2017) 10:10–7. 10.4103/0974-1208.204018 PMC5405641 28479750

[33] Potti A Bild A Dressman HK Lewis DA Nevins JR Ortel TL. Gene-Expression Patterns Predict Phenotypes of Immune-Mediated Thrombosis. Blood (2006) 107:1391–6. 10.1182/blood-2005-07-2669 PMC1895419 16263789

[34] de Jesus GR Sciascia S Andrade D Barbhaiya M Tektonidou M Banzato A. Factors Associated With First Thrombosis in Patients Presenting With Obstetric Antiphospholipid Syndrome (APS) in the APS Alliance for Clinical Trials and International Networking Clinical Database and Repository: A Retrospective Study. BJOG (2019) 126:656–61. 10.1111/1471-0528.15469 PMC7382947 30222236

