# Supplementary material for: Urine Proteomics Differentiate Primary Thrombotic Antiphospholipid Syndrome From Obstetric Antiphospholipid Syndrome
Source: Front Immunol. 2021 Aug 19;12:702425. doi: 10.3389/fimmu.2021.702425 (PMC8416615; doi:10.3389/fimmu.2021.702425)
Supplement: Supplementary file 1 [file DataSheet_1.docx]

**Supplementary Table 1. The numbers of proteins analyzed by iTRAQ study among patients with TAPS, patients with OAPS and HCs.**

| Comparison | Up-regulated proteins | Down-regulated  proteins | Total proteins |
| --- | --- | --- | --- |
| TAPS vs HCs | 36 | 392 | 428 |
| OAPS vs HCs | 132 | 337 | 469 |
| OAPS vs TAPS | 349 | 147 | 496 |

iTRAQ, isobaric tags for relative and absolute quantification; TAPS, thrombotic antiphospholipid syndrome; OAPS, obstetric antiphospholipid syndrome; HCs, healthy controls.

**Supplementary Table 2. The representative 10 secreted proteins in the urine proteomics of patients with OAPS vs. patients with TAPS.**

| Gene | Protein | Protein function | Fold change | P value |
| --- | --- | --- | --- | --- |
| FBN2 | Fibrillin-2 | Regulate the early process of elastic fiber assembly | 2.452 | 0.029 |
| ECRG4 | Augurin | Attenuate cell proliferation and induce senescence | 2.334 | <0.001 |
| C4BPB | C4b-binding protein beta chain | Controls the classical pathway of complement activation | 2.122 | 0.028 |
| ADM | ADM | adrenomedullin receptor binding, hormone activity, signaling receptor binding | 2.053 | 0.002 |
| PDGFB | Platelet-derived growth factor subunit B | Regulation of cell proliferation, cell migration, survival and chemotaxis | 1.991 | 0.003 |
| APOA4 | Apolipoprotein A-IV | major component of HDL and chylomicrons | 0.581 | 0.007 |
| IGLV5-37 | Immunoglobulin lambda variable 5-37 | adaptive immune response, immune response | 0.543 | 0.041 |
| C8G | Complement component C8 gamma chain | constituent of the membrane attack complex | 0.506 | 0.008 |
| CXCL12 | Stromal cell-derived factor 1 | Chemoattractant active on T-lymphocytes and monocytes | 0.472 | 0.045 |
| LBP | Lipopolysaccharide-binding protein | Acts as an affinity enhancer for CD14 | 0.406 | 0.036 |

TAPS, thrombotic antiphospholipid syndrome; OAPS, obstetric antiphospholipid syndrome.

**Supplementary Table 3. The representative 10 secreted proteins in the urine proteomics of patients with TAPS vs. HCs.**

| Gene | Protein | Protein function | Fold change | P value |
| --- | --- | --- | --- | --- |
| CXCL12 | Stromal cell-derived factor 1 | Chemoattractant active on T-lymphocytes and monocytes | 2.192 | 0.033 |
| C8G | Complement component C8 gamma chain | A constituent of the membrane attack complex | 2.039 | 0.008 |
| CELA2A | Chymotrypsin-like elastase family member 2A | Acts upon elastin | 1.981 | 0.042 |
| ZG16B | Zymogen granule protein 16 homolog B | Carbohydrate binding | 1.879 | 0.002 |
| MMP3 | Stromelysin-1 | Degrade fibronectin, laminin and gelatins | 1.493 | 0.019 |
| RNASE4 | Ribonuclease 4 | marked specificity towards the 3' side of uridine nucleotides | 1.440 | 0.021 |
| PDGFRA | Platelet-derived growth factor receptor alpha | Regulate embryonic development, cell proliferation, survival and chemotaxis | 0.527 | 0.025 |
| COX2 | Cytochrome c oxidase subunit 2 | Component of the cytochrome c oxidase | 0.515 | 0.022 |
|  |  |  |  |  |
| SMOC2 | SPARC-related modular calcium-binding protein 2 | Transcription factor that may act during endoplasmic reticulum | 0.502 | 0.049 |
| PROZ | Vitamin K-dependent protein Z variant 1 | Assist hemostasis | 0.500 | 0.034 |

TAPS, thrombotic antiphospholipid syndrome; HCs, healthy controls.

**Supplementary Table 4. The representative 10 secreted proteins in the urine proteomics of patients with OAPS vs. HCs.**

| Gene | Protein | Protein function | Fold change | P value |
| --- | --- | --- | --- | --- |
| PPBP | Platelet basic protein | Stimulates DNA synthesis, mitosis, glycolysis | 2.408 | 0.002 |
| CLPS | Colipase | A cofactor of pancreatic lipase | 2.167 | 0.008 |
| CELA2A | Chymotrypsin-like elastase family member 2A | Acts upon elastin | 2.085 | 0.018 |
| PDGFB | Platelet-derived growth factor subunit B | Regulation of cell proliferation, cell migration, survival and chemotaxis | 1.804 | 0.004 |
| PILRA | Paired immunoglobulin-like type 2 receptor alpha | A cellular signaling inhibitory receptor | 1.627 | 0.011 |
| MMP3 | Stromelysin-1 | Degrade fibronectin, laminin, gelatins | 1.531 | 0.012 |
| ACE | Angiotensin-converting enzyme | increase of the vasoconstrictor activity of angiotensin | 0.778 | 0.005 |
| SOD3 | Extracellular superoxide dismutase [Cu-Zn] | Protect the extracellular space from toxic effect of reactive oxygen intermediates | 0.773 | 0.029 |
|  |  |  |  |  |
| IL7R | Interleukin-7 receptor subunit alpha | Receptor for interleukin-7, receptor for thymic stromal lymphopoietin (TSLP). | 0.769 | 0.009 |
| PDGFRA | Platelet-derived growth factor receptor alpha | Regulate embryonic development, cell proliferation, survival and chemotaxis | 0.552 | 0.021 |

OAPS, obstetric antiphospholipid syndrome; HCs, healthy controls.

**Supplementary Figure 1.** **Venn diagram of changed proteins among patients with OAPS, patients with TAPS and HCs analyzed by iTRAQ proteomics.**

**Supplementary Figure 2. Validation of matrix metalloproteinase 3 (MMP3) and platelet-derived growth factor receptor alpha (PDGFRA) as urinary biomarkers for patients with OAPS and patients with TAPS.** A. MMP3 levels in the urine of patients with OAPS, patients with TAPS and HCs. B. PDGFRA levels in the urine of patients with OAPS, patients with TAPS and HCs. ***p*<0.01.

**Supplementary Figure 3. Levels of urinary CXCL12 and PDGFB were analyzed according to the number of positive aPL antibodies in patients with OAPS and patients with TAPS.** A. CXCL12 levels in patients with OAPS with single, double, and triple aPLs. B. PDGFB levels in patients with OAPS with single, double, and triple aPLs. C. CXCL12 levels in patients with TAPS with single, double, and triple aPLs. D. PDGFB levels in patients with TAPS with single, double, and triple aPLs. Single, double, and triple refers to the positive numbers of aPLs.

**Supplementary Figure 4. Levels of urinary CXCL12 and PDGFB were analyzed according to the numbers of adverse pregnancy outcomes in patients with OAPS.** A. CXCL12 levels in patients with one miscarriage and more than one miscarriage. B. CXCL12 levels in patients with one intrauterine death and more than one intrauterine death. C. PDGFB levels in patients with one miscarriage and more than one miscarriage. D. PDGFB levels in patients with one intrauterine death and more than one intrauterine death.

**Supplementary Figure 5. Levels of urinary CXCL12 and PDGFB grouped according to type of thrombosis in patients with TAPS.** A. CXCL12 levels in patients with arterial thrombosis and without arterial thrombosis. B. PDGFB levels in patients with arterial thrombosis and without arterial thrombosis. C. CXCL12 levels in patients with one arterial thrombosis and more than one arterial thrombosis. D. PDGFB levels in patients with one arterial thrombosis and more than one arterial thrombosis.

**Supplementary Figure 6. Levels of urinary CXCL12 and PDGFB** **between males and females** **in patients with TAPS.** A. CXCL12 levels in males and females in patients with TAPS. B. PDGFB levels in males and females in patients with TAPS.
