# Supplementary figures and images for: Urine Proteomics Differentiate Primary Thrombotic Antiphospholipid Syndrome From Obstetric Antiphospholipid Syndrome
Source: Front Immunol. 2021 Aug 19;12:702425. doi: 10.3389/fimmu.2021.702425 (PMC8416615; doi:10.3389/fimmu.2021.702425)

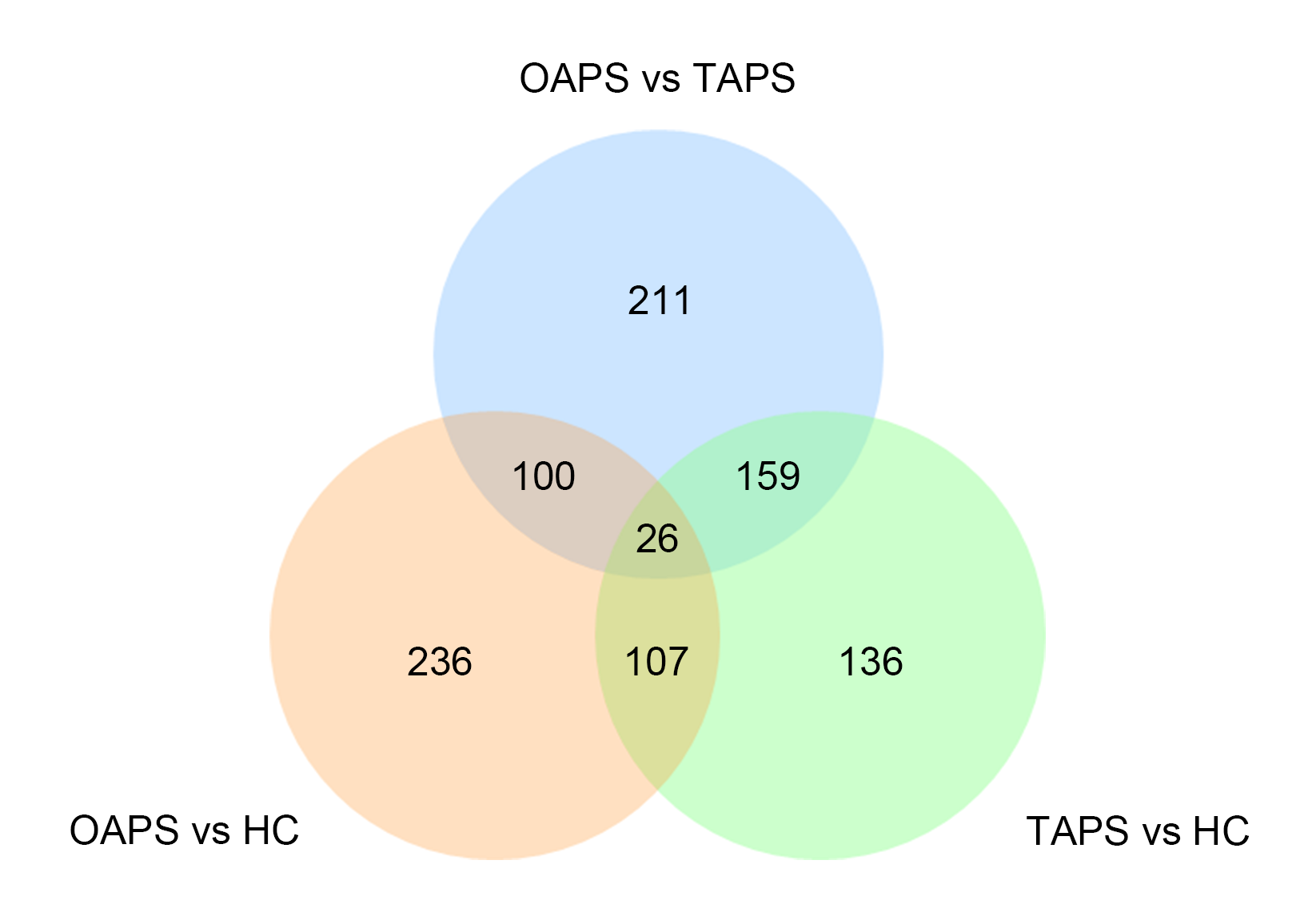

Supplement: Supplementary file 2 [file Image_1.tif]

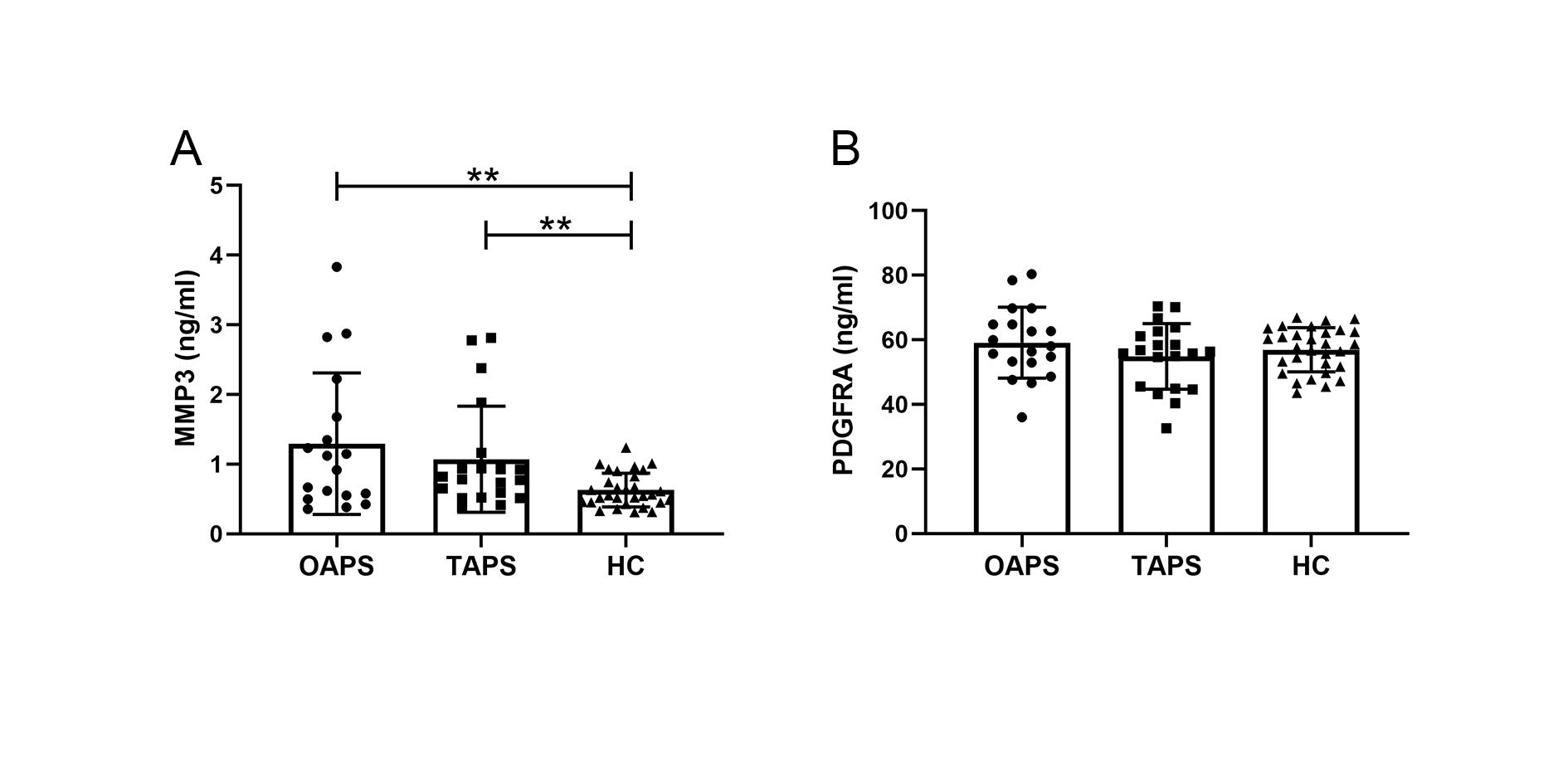

Supplement: Supplementary file 3 [file Image_2.tif]

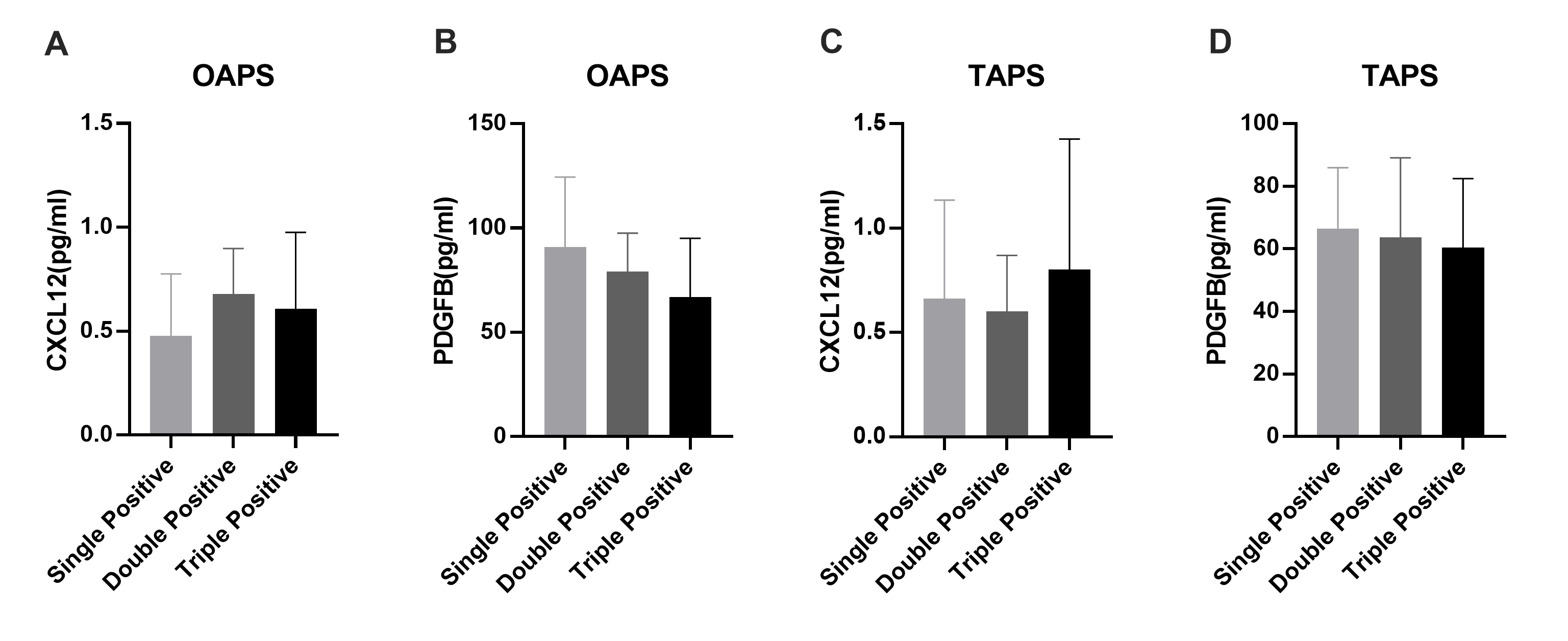

Supplement: Supplementary file 4 [file Image_3.tif]

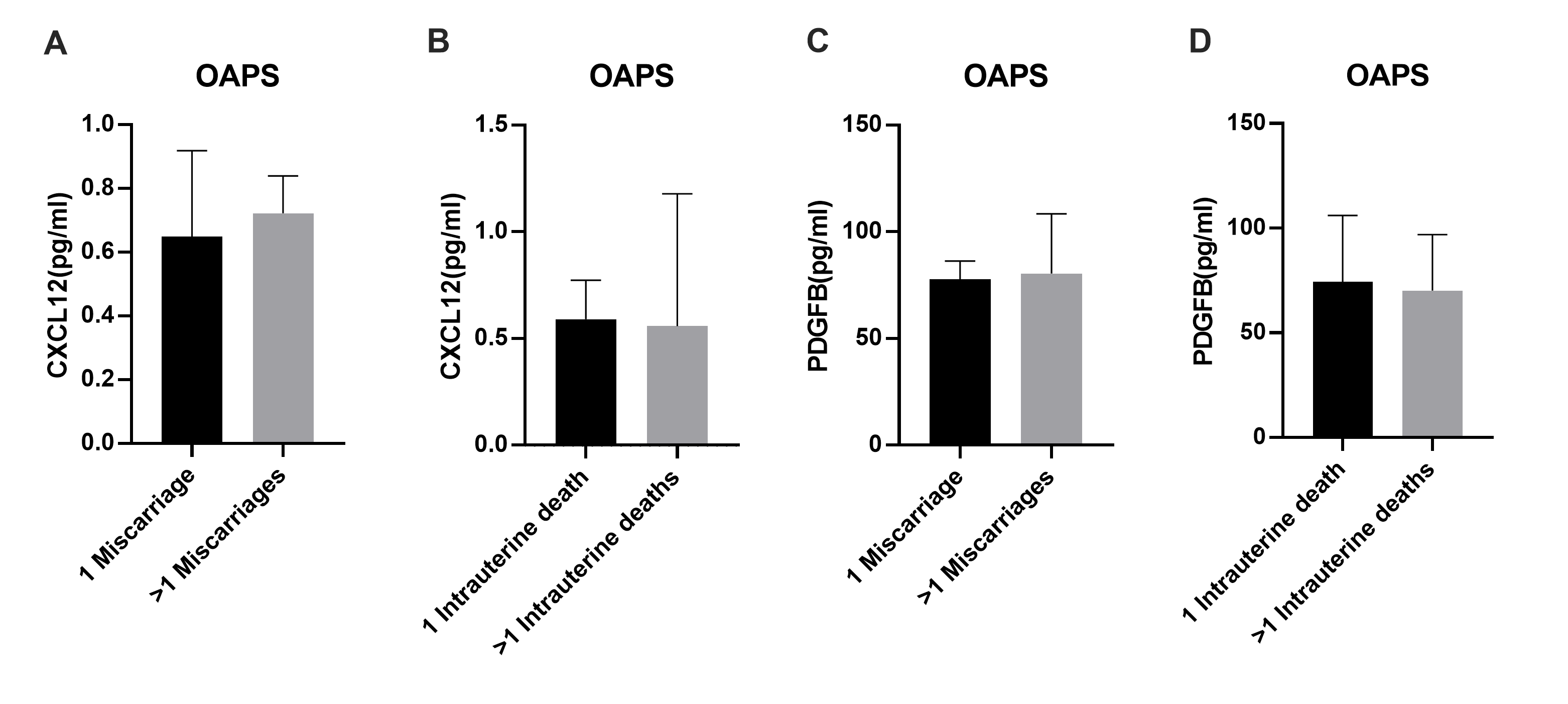

Supplement: Supplementary file 5 [file Image_4.tif]

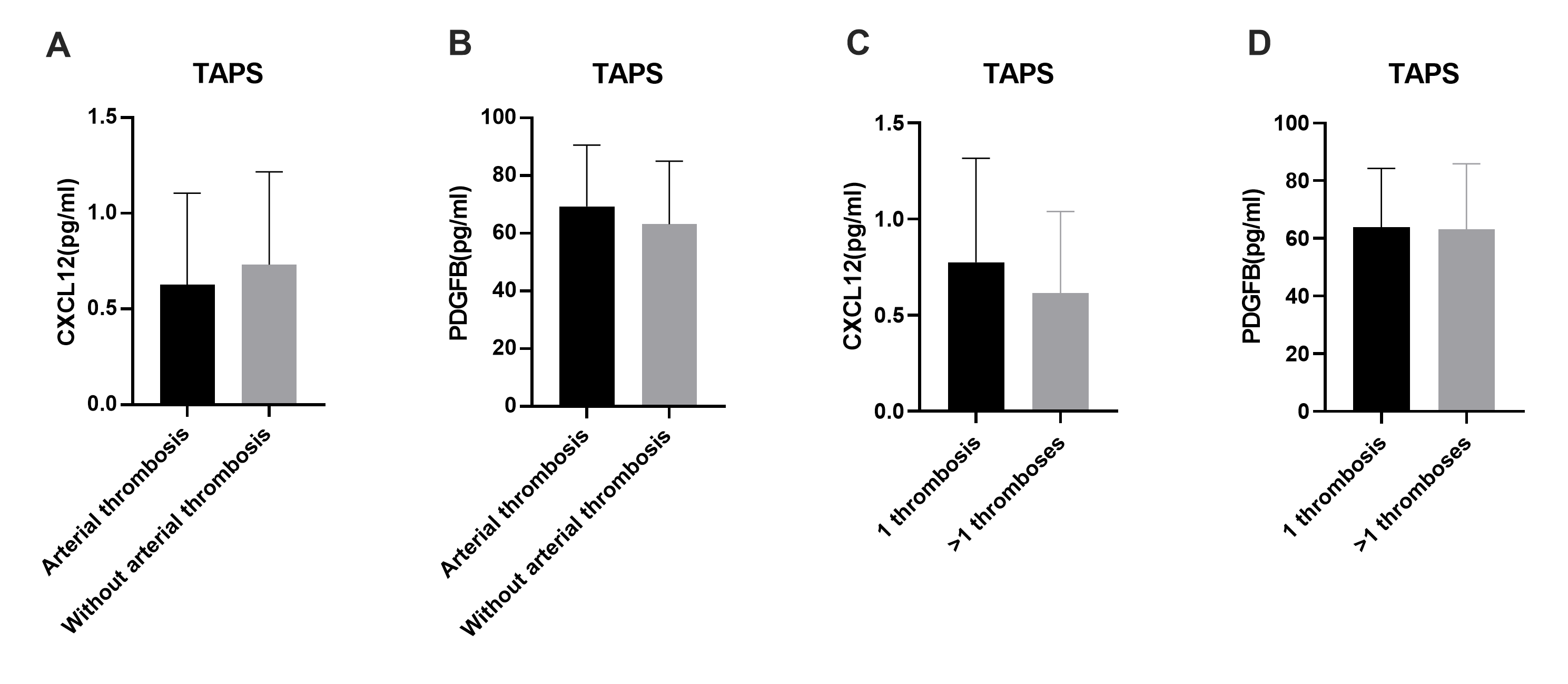

Supplement: Supplementary file 6 [file Image_5.tif]

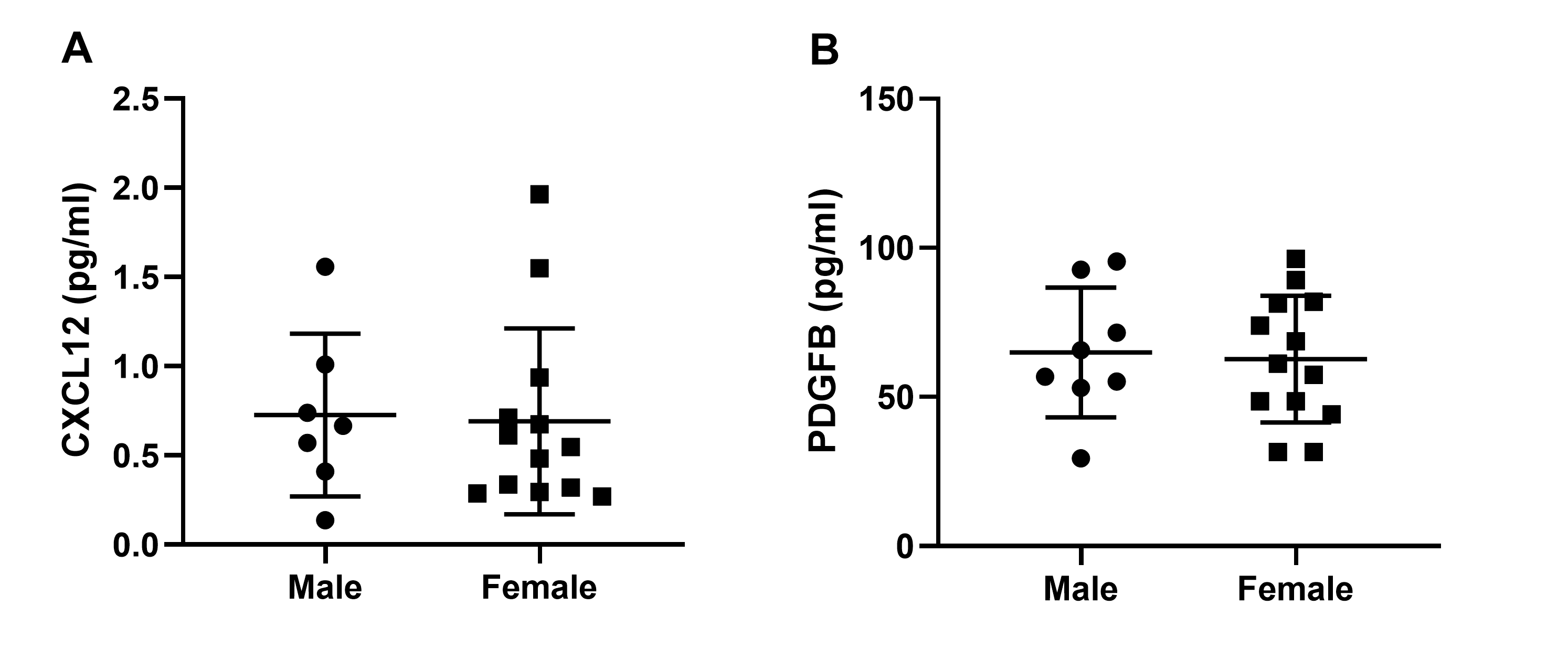

Supplement: Supplementary file 7 [file Image_6.tif]
